# Early developmental exposures shape trade-offs between acquired and innate immunity in humans

**DOI:** 10.1093/emph/eow022

**Published:** 2016-08-15

**Authors:** Alexander V. Georgiev, Christopher W. Kuzawa, Thomas W. McDade

**Affiliations:** Department of Anthropology, Northwestern University, Evanston, IL 60208, USA

**Keywords:** ecoimmunology, innate immunity, C-reactive protein, Epstein–Barr virus antibodies, life history theory

## Abstract

**Background and objectives:**

Life history theory predicts resource allocation trade-offs between competing functions and processes. We test the hypothesis that relative investment towards innate versus acquired immunity in humans is subject to such trade-offs and that three types of early developmental exposures are particularly salient in shaping adult immunophenotype: (i) pathogen exposure, (ii) nutritional resources; and (iii) extrinsic mortality cues.

**Methodology:**

We quantified one aspect each of innate and acquired immune function, via C-reactive protein and Epstein–Barr virus antibodies, respectively, in a sample of 1248 men and women from the Philippines (ca. 21.5 years old). Early developmental exposures were assessed via long-term data collected prospectively since participants’ birth (1983–4). We calculated a standardized ratio to assess relative bias towards acquired versus innate immune function and examined its relationship to a suite of predictors via multiple regression.

**Results:**

In partial support of our predictions, some of the measures of higher pathogen exposure, greater availability of nutritional resources, and lower extrinsic mortality cues in early life were associated with a bias toward acquired immunity in both men and women. The immune profile of women, in particular, appeared to be more sensitive to early life pathogen exposures than those of men. Finally, contrary to prediction, women exhibited a greater relative investment toward innate, not acquired, immunity.

**Conclusions and implications:**

Early environments can exert considerable influence on the development of immunity. They affect trade-offs between innate and acquired immunity, which show adaptive plasticity and may differ in their influence in men and women.

## Abstract (precis)

We tested the hypothesis that early-life developmental exposures (nutrition, pathogens, and cues of extrinsic mortality) affect the balance of investment between acquired and innate immune defenses in adults. Analyses of two immuno-markers suggest greater nutrition and pathogen exposure, and lower extrinsic mortality cues associated with a small bias towards acquired defenses.

## Background and Objectives

Individual differences in immune function have received considerable public health and clinical attention owing to the rising prevalence of chronic inflammation and atopic diseases in affluent industrialized populations [1–3], but a more complete understanding of this variation should also incorporate an evolutionary life history perspective [4–6]. The key principle of this perspective is the notion that any investment in immune defenses is costly [7, 8] and diverts resources from allocations to other physiological or developmental processes [9, 10]. This leads to trade-offs in investment between immunity and other functions, especially when resources are limited. Additionally, a life history approach views biology through the lens of the lifecycle and thus also focuses attention on development and the plasticity involved in calibrating the phenotype of the individual, in response to inputs from the environment early in life [11].

These threads converge within the emerging discipline of ecological immunology (ecoimmunology), which places particular emphasis on how ecological factors affect an individual’s immunophenotype and thus places an organism’s investment in immune defenses within the context of local environmental conditions [12, 13]. Such an ecoevolutionary approach aims to explain substantial species- and individual-level variation in immune function and to provide a mechanistic understanding of the life-history trade-offs individuals may incur over their lifetime. A recent focus of research in ecoimmunology has been examining and explaining the trade-offs within the immune system, specifically between its innate and acquired branches [12, 14–20]. The explanatory frameworks developed within the literature on other vertebrates, however, have not been explicitly tested on humans.

Previous work in human ecoimmunology has focused primarily on trade-offs between overall investment toward immune function and other energetically costly processes such as growth and reproduction [4, 21–25]. Trade-offs can also occur within the immune system, which comprised two main branches: innate, or non-specific immunity, and acquired, or specific, immunity. Innate immunity acts against a broad variety of pathogens and includes anatomical barriers (skin and mucosal membranes), antimicrobial soluble proteins (e.g. complement, lysozyme), natural killer cells, and phagocytic cells. Inflammation, driven by acute phase proteins and phagocytic cells, in particular, is a key mechanism of the innate immune response. Conversely, acquired defenses, primarily via the action of T and B lymphocytes, are highly specific to particular pathogens and form the core of immunological memory following an initial exposure to a pathogen. Some immune defenses (e.g. some of the antimicrobial peptides), however, have dual roles, providing both non-specific and specific response to pathogens [26], illustrating the close integration of the two branches of the immune system

The costs and benefits of subsystems of immune defense are variable, with the optimal allocation of resources across different arms of immunity depending on the specifics of the local ecology [14, 27–29]. The branches typically work in coordination to provide effective defenses against pathogens and parasites, but the relative balance of investment in innate versus acquired immunity varies greatly within and across species [15–17]. Innate immune defenses are relatively “cheaper” to develop and can provide short-term fitness benefits but incur higher costs when utilized (e.g. oxidative stress and tissue damage) and can thus be generally less cost-effective than acquired defenses. Conversely, acquired defenses involve significantly higher up-front developmental costs but are more efficient and less costly to deploy later in life once developed, with significant fitness benefits being realized in the long term [27, 28]. Building on past work in other species [14, 15, 30], we recently proposed that the balance of investment in innate versus acquired immune defenses in humans will depend upon three axes of ecological variation experienced early in development: infectious exposure, nutritional resources, and extrinsic mortality cues [31].

First, we predict that individuals growing up in a high-pathogen environment will prioritize investing in specific, efficient acquired immune defenses, all else being equal. The long-term benefits of investing in development of acquired immunity will increase with the frequency of encountering pathogens repeatedly in one’s environment. Conversely, if early life pathogen exposure is low, this would reduce the potential long-term payoffs of investing in costly development of acquired immunity and favor an immune profile biased toward non-specific defenses [31].

Second, we predict that resource availability will also affect the optimal investment to the two arms of the immune system. Although specific, acquired immune defenses provide clear advantages in a high-pathogen environment, the organism’s ability to invest in specific immune development can be constrained if nutritional resources during early development are scarce. Thus, we predict that greater resource abundance in the prenatal and early post-natal period will allow increased investment in acquired immunity, given its relatively high developmental costs. Conversely, if early-life nutritional resources are limited, this will constrain the organism’s ability to invest in the development of acquired immunity. Later in life, such an individual will exhibit an immunophenotype that relies to a greater extent on innate, rather than acquired, defenses, even if nutritional conditions in adulthood are more favorable than during the developmentally critical early-life period when we predict that biases in immune allocation are set [31].

Finally, we predict that as the potential risk of unavoidable (extrinsic) mortality increases, the optimal pattern of immune investment will shift to favor non-specific immunity. Under such conditions, the high upfront costs of acquired immunity would receive a lower priority, as shorter lifespan reduces the long-term benefits of acquired defenses. Conditions that may signal higher risk of extrinsic mortality (e.g. experiences of psycho-social adversity such as parental absence or sibling death) would therefore be associated with a shift in resource allocation toward immune function prioritizing the less costly in the short-term option, i.e. innate immunity. Cues of extrinsic mortality have been shown to shift developmental priorities of investment in other domains, e.g. from investment in somatic growth to early reproduction [32–35], and we predict that similar optimization switches may also occur at the level of immune sub-systems. Extending this logic, given the lower average life expectancies among men in nearly all human populations, compared to women, we also predict that men would invest relatively less toward acquired than innate immune defenses, in comparison to women [31].

We test these predictions using data from a prospective longitudinal study of humans in the Philippines. Using two markers of immune function, C-reactive protein (CRP) and Epstein–Barr virus (EBV) antibodies, we assess relative investment toward innate and acquired immune defenses, respectively, in a large cohort of ca. 21.5-year olds. With data on their early-life environments (pathogen exposure, nutritional condition, and psycho-social adversity), we examine whether their adult immunophenotype exhibits patterns consistent with the hypothesis of early developmental plasticity and trade-offs across branches of immune function [31]. To place any trade-offs within the immune system in a broader context, we also examine whether any of the early-life exposure factors predicted to affect relative investment toward acquired and innate immunity also affected overall levels of immunocompetence.

## Methods

### Participants and data collection

The Cebu Longitudinal Health and Nutrition Survey (CLHNS) began in 1983–4 with the recruitment of 3327 pregnant women from randomly selected urban and rural neighborhoods in Metropolitan Cebu City, Philippines [36]. The study of their offspring included detailed bimonthly surveys for 2 years after birth to assess infectious morbidity, growth, and other characteristics, as well as several subsequent follow-up surveys across childhood, adolescence, and into adulthood. The analyses presented here focus on 1479 offspring for whom complete anthropometric, interview, and biomarker data were available from the 2005 survey [37]. Women who were pregnant during the survey were excluded from the analyses, as CRP has been shown to be elevated during gestation in this population [38], resulting in a sample of *N* = 1453. Because one of our outcomes (CRP) is elevated acutely during infections, participants reporting infectious symptoms during the interview were also excluded (*N* = 205). Median CRP was 0.7 mg/L for the participants reporting infectious symptoms, compared with 0.2 mg/l for the rest of the sample. Although menstrual phase is also known to affect CRP levels in women [25], preliminary analysis of self-report interview data of last menstrual period did not reveal significant differences in CRP across phases of the cycle. Specifically, for a subset of currently menstruating women for which we could ascertain their position in the menstrual cycle at the time of blood sampling from self-report data (*N* = 414), we did not find significant differences in CRP levels between women in the early follicular, late follicular, or luteal phase of the cycle (Kruskal–Wallis test, χ^2 ^= 0.321, df = 2, *P* = 0.852). A total of 1248 healthy, non-pregnant individuals (men and women) contributed data to these analyses. All data were collected under conditions of written informed consent using protocols approved by the Institutional Review Board of the University of North Carolina, Chapel Hill. Data from the CLHNS are publically available from the Dataverse Network (a digital repository) of the Odum Institute at the University of North Carolina, Chapel Hill: http://arc.irss.unc.edu/dvn/dv/cebu.

### Quantifying adult immunophenotype

To assess the balance of investment toward innate versus acquired immunity, we measured two biomarkers of immune function: CRP (innate) and EBV antibodies (acquired immunity). CRP is an acute phase protein and biomarker of inflammation and an important component of innate immunity. Produced primarily by hepatocytes in response to cytokine signals, CRP activates complement, promotes activity of phagocytic cells, and opsonizes bacteria, fungi, and parasites [39]. Concentrations of CRP increase rapidly in response to a wide range of infectious agents, further underscoring its role in non-specific anti-pathogen defenses. However, inflammatory processes contribute to the pathophysiology of atherosclerosis and chronically elevated concentrations of CRP have been associated with elevated risk for cardiovascular disease, type 2 diabetes, late-life disability, and all-cause mortality in industrialized populations [40]. CRP, therefore, can be interpreted as a marker of investment in innate immune defenses that protect against infectious disease, but with the potential for long-term costs associated with increased risk for diseases of aging. CRP concentrations were determined in plasma, as described previously [37].

The EBV is a ubiquitous herpesvirus that infects nearly 90% of adults in industrialized nations, while infection rates approach 100% during the first 5 years of life in lower income nations [41, 42]. Once infected, individuals permanently harbor EBV, and adequate cell-mediated immune function is critical for maintaining the virus in a latent state: immunosuppression allows EBV to reactivate and release viral antigens into circulation, to which a humoral antibody response may emerge [43]. Increases in antibodies specific to EBV are associated with concurrent reductions in memory T-cell proliferation and cytotoxic T-cell killing of infected cells [44, 45]. Antibodies against EBV can therefore be interpreted as an indirect measure of investment in specific, cell-mediated immune defenses. EBV antibodies were quantified in dried blood spot samples [46, 47].

Across all individuals, CRP levels were not significantly correlated with EBV antibody titers (*r* = 0.03, *N* = 1248, *P* = 0.28). To evaluate the relative investment toward innate versus acquired immunity, we constructed a ratio between the two variables. Because 36.1% of samples (*N* = 450) had CRP levels below assay sensitivity, we re-coded all zero values as 0.1. We then log-10 transformed and standardized (mean = 10, SD = 1) the values of CRP and EBV to allow a direct comparison. Because higher levels of EBV antibodies indicate *reduced* cell-mediated immune function, we multiplied log-EBV antibody titers by −1 (i.e. reverse-scoring) so that higher values would indicate higher levels of cell-mediated immunity. We then calculated the ratio of standardized acquired (cell-mediated) immunity (based on EBV values) to standardized innate immunity (based on CRP values) as the CMI:CRP ratio. A value of 1.0 represents equal investments in both aspects of immunity, relative to the sample distributions. Higher values represent bias toward acquired immunity, while lower values represent a bias toward innate immunity.

To evaluate the intra-individual consistency/tracking (within the population) of our measures of innate and acquired immunity, we compared immune responses in a sub-sample of study participants, measured at two time points. Concentrations of CRP were measured during adolescence (14–15 years of age) and young adulthood (21–22 years of age) in a subsample of study participants (*N* = 341). These CRP values were positively and significantly correlated (*r* = 0.24, *P* < 0.001). Even though the correlation was not particularly strong, these data nevertheless suggest that over time inter-individual differences in CRP remained somewhat consistent. At the age 14–15 years, a subset of participants received a typhoid vaccine, and 47.9% of the sample responded to vaccination with a ≥4-fold increase in anti-typhoid antibody titer [48]. Robust antibody response to vaccination reflects increased levels of acquired immune defenses. At age 21–22 years, EBV antibody titers were higher in the group of non-responders (mean = 1.91, SD = 0.26, *N* = 36), indicating reduced cell-mediated immunity. Vaccine responders had lower levels of EBV antibodies (mean = 1.81, SD = 0.24, *N* = 37), indicating higher cell-mediated immunity. The difference in EBV antibodies across the groups approaches significance (*t* = 1.75, *N* = 73, *P* = 0.08) and is consistent with the interpretation that individual differences in overall investments in acquired immune defenses are relatively stable in this sample.

### Modeling variation in immunophenotype

To model inter-individual variation in CMI:CRP ratios, we considered three main groups of factors: pathogen exposure, nutrition, and early life psychosocial adversity. We used three variables to characterize childhood pathogen exposure [49]. First, we calculated the total number of bi-monthly surveys conducted in the first 2 years of life, during which infants displayed symptoms of diarrhea or respiratory infection in the week preceding the interview, as reported by their mothers. Second, we summed the number of bi-monthly surveys, during the first year of life, when the infant was observed crawling on the floor and animals were present in the home, as a proxy measure of the likelihood of exposure to animal feces [49]. Third, we classified infants depending on the month of their birth as dry and wet season born. Being born during the dry season in this population is a predictor of higher pathogen exposure as these infants spend a greater proportion of their first year of life in the wet season, which is a period of higher microbial exposure [49]. We used infant birth weight and the rate of weight gain between birth and 2 years of age as markers of pre-natal and early post-natal nutritional sufficiency, respectively [50]. To assess early life adversity, we considered three measures of psychosocial risk in childhood that are commonly assumed to signal instability in caregiving relationships and increased mortality risk [51, 52]. As described previously [53], we constructed three dichotomous variables to indicate (i) maternal absence (if subjects lived in a separate household than their mother at 8.5 or 11.5 years of age); (ii) paternal instability up to ca. 11.5 years old (father deceased or absent, mother unmarried in first year of child’s life or beyond, or mother remarried); and (iii) whether participants experienced the death of a sibling during the same period.

### Statistical analysis

We carried out linear regression analysis in R 3.0.3 [54] with the CMI:CRP standardized ratio as the dependent variable and ten independent variables. Eight of those were measures of early life developmental exposures (1. birthweight, 2. weight gain in the first 2 years, 3. incidence of infectious morbidity, 4. likelihood of exposure to animal feces, 5. season of birth (dry/wet), 6. maternal absence, 7. paternal instability, 8. sibling death) and two of them were control factors (9. total immune investment score and 10. waist circumference). We included the two “control factors” in the model to adjust for individual differences in traits that could confound associations with early developmental factors. We included total immune investment (the sum of standardized innate and acquired immune scores) in the model as we expected that if overall investment toward immune function were greater, the balance between investment toward acquired and innate immunity would shift toward the more developmentally costly arm (acquired). Adult waist circumference (measured during the in-home interview in 2005 using standard procedures) was included because adiposity is an important and potentially independent influence on CRP levels [37].

Given known sex-specific patterns in immune development [55] and previous evidence of sex differences in the physiology of our study cohort [56–58], we also tested for sex differences in the role of early life factors on shaping adult immune function. To do so, we first constructed a maximal model, which included the interactions of sex with all other predictors of interest (excluding the control variables). To interpret the effects of factors that were not involved in a significant interaction with sex, we then excluded all non-significant interactions to obtain a more parsimonious model containing all predictors and only significant interactions. Significant interactions were interpreted via post hoc Tukey contrasts using the function glht in the R package multcomp [59].

Initially, we also explored the role of three measures of socioeconomic status (SES), assessed both at birth (for the mother) and at the age of sample collection in young adulthood (level of maternal education, a score of household assets and adjusted household income), as described previously [60]. Because none of the SES measures predicted the CMI:CRP ratio (all *P* > 0.4) and including them reduced overall model fit, we did not consider them further in analyses.

We conducted model validation by checking for multicollinearity among predictor variables (variance inflation factors; package “car”: [61]) and by visually inspecting model diagnostic plots. Variance inflation factors for individual predictors ranged between 1.01 and 1.80, suggesting that multicollineariy was not an issue. Model diagnostic plots did not indicate severe heteroskedasticity.

## Results

We first considered whether infectious, nutritional, and extrinsic mortality risk variables predicted the total level of immune investment (sum of the CMI and CRP standardized scores). Males had significantly higher values than females (*b* = 0.26, SE = 0.08, *P* = 0.002), but early developmental exposures were not associated with total immune investment (all *P* > 0.19; results not shown).

There was considerable inter-individual variation in CMI:CRP scores, with values ranging from 0.61 (indicating 39% higher CRP relative to CMI) to 1.36 (indicating 36% higher CMI relative to CRP; Table 1). CMI and CRP were weakly negatively associated among female subjects (*r* = −0.08, *N* = 539, *P* = 0.067), but no such relationship was present among males (*r* = 0.0007, *N* = 709, *P* = 0.986). Females also had significantly lower CMI:CRP ratio than males (Table 1) suggesting, contrary to one of our predictions, an overall female investment bias toward innate immunity.

**Table 1.** Sample characteristics (*N* = 1248 individuals)

Variable	Women (*N* = 539)		Men (*N* = 709)			
	Mean (SD)/%	Range	Mean (SD)/%	Range	*t*-statistic/ z-statistic^a^	*P* value
**Immune measures**						
EBV antibodies (ELISA units)	98.68 (58.68)	13.95–300.34	77.64 (48.82)	16.60–286.18	−6.74	<0.0001
CRP (mg/L)	1.42 (4.84)	0.10–79.00	1.16 (3.71)	0.10–49.3	−1.04	0.297
CMI:CRP (standardized ratio)	0.99 (0.14)	0.61–1.36	1.03 (0.13)	0.63–1.34	4.95	<0.0001
Total (CMI + CRP) immune investment	19.80 (1.39)	16.98–24.72	20.15(1.37)	16.97–24.47	4.43	<0.0001
**Independent variables**						
Age in 2005 (years)	21.47 (0.33)	20.76–22.44	21.47 (0.30)	20.8–22.09	0.13	0.900
Birth weight (kg)	2.99 (0.41)	1.7–4.1	3.02 (0.43)	0.91–4.20	1.04	0.300
Weight gain, 0–2 years (kg)	6.48 (1.03)	2.77–9.1	7.10 (1.10)	2.8–11.04	10.30	<0.0001
Infectious morbidity, 0–2 years (no. of episodes)	9.00 (2.10)	2–12	9.22 (2.09)	1–12	1.89	0.059
Likelihood of exposure to animal feces, 6–12 months (no. of intervals)	1.18 (1.24)	0–4	1.32 (1.31)	0–4	1.88	0.061
Waist circumference, 2005 (cm)	68.19 (7.37)	53.9–104	72.10 (7.54)	56.5–112	9.14	<0.0001
Born in the dry season (%)	20.96		20.45		−0.22	0.824
Experienced maternal absence (%)	8.35		9.73		0.84	0.401
Experienced sibling death (%)	13.17		12.83		−0.1758	0.861
Experienced paternal instability (%)	8.35		8.46		0.07	0.943

a T-test-unequal variances with Welch's correction; z-statistic for two-sample test of proportions.

**Figure 1.**
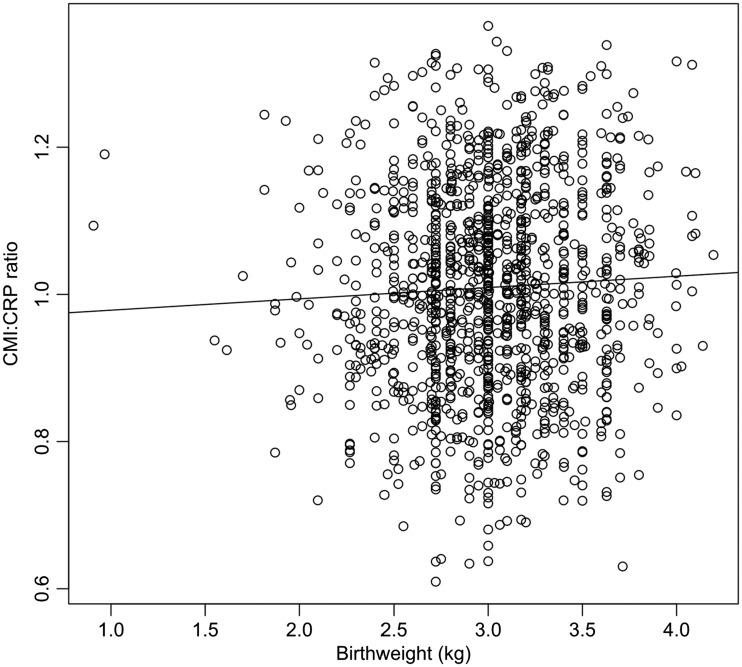
Relationship between birthweight and CMI:CRP ratio in our dataset (*N* = 1248). Regression line based on a simple linear model, for illustrative purposes only (statistical details are shown in Table 2)

**Figure 2.**
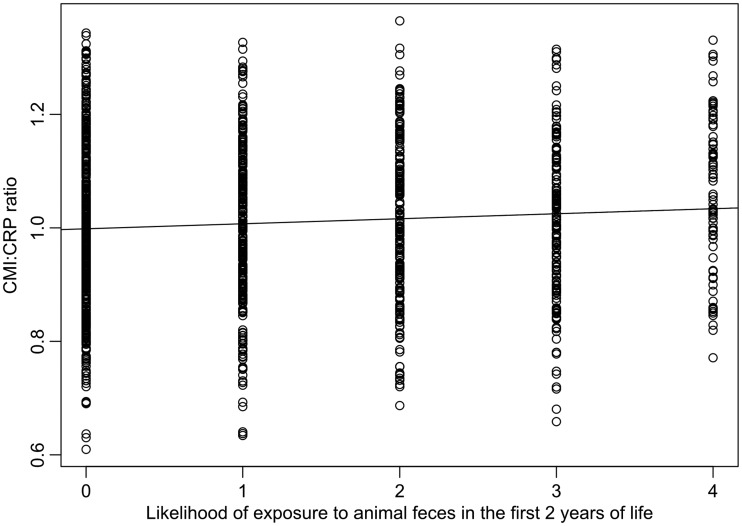
Relationship between likelihood of exposure to animal feces in the first year of life and CMI:CRP ratio in our data set (*N* = 1248). Regression line based on a simple linear model, for illustrative purposes only (statistical details are shown in Table 2)

**Figure 3.**
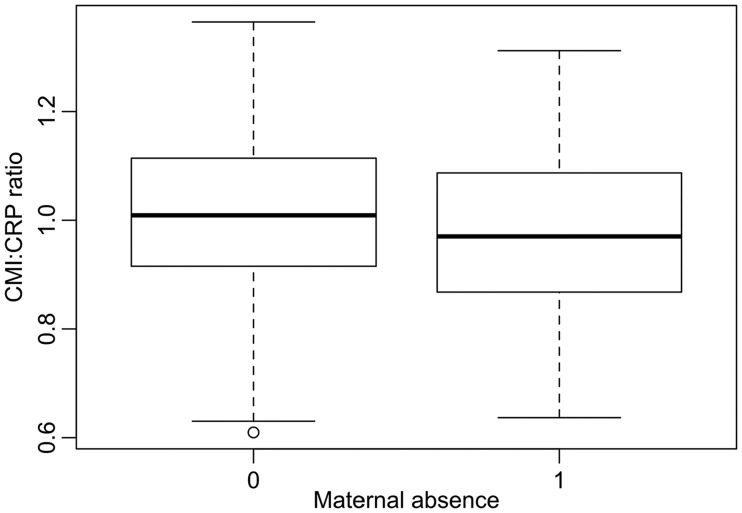
Difference in CMI:CRP ratio between individuals who experienced maternal absence and those who did not. Boxplots show the median (thick line), the inter-quartile range (box), range (whiskers), excluding outliers (open circles, over 1.5 times the interquartile range from the box) (statistical details are shown in Table 2)

**Figure 4.**
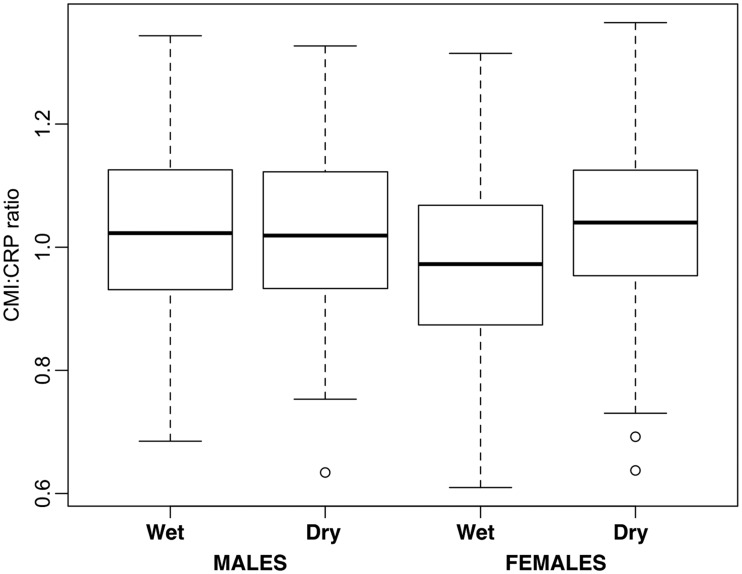
Difference in CMI:CRP ratio between individuals born in the dry and in the wet season (for men and women). Boxplots show the median (thick line), the inter-quartile range (box), range (whiskers), excluding outliers (open circles, over 1.5 times the interquartile range from the box) (statistical details are shown in Table 2)

In our model of CMI:CRP ratio variation, of all early-life exposures, only one (season of birth) was involved in a significant interaction with sex (maximal model: *F*_19 1228 _= 5.49, Adj. *R*^2 ^= 0.064, *P* < 0.0001). After excluding all non-significant interactions, we obtained a more parsimonious model (*F*_12 1235 _= 7.95, Adj. *R*^2 ^= 0.063, *P* < 0001, Table 2). In line with predictions, higher birth weight (Fig. 1) and greater likelihood of exposure to animal feces in the first year of life (Fig. 2) were associated with higher a CMI:CRP ratio in later life, whereas maternal absence (Fig. 3) was associated with lower CMI:CRP ratio (Table 2). Sex and season of birth were involved in a significant interaction such that females (post-hoc Tukey contrasts: *b* = −0.06, SE 0.01, *P* < 0.001) but not males (b = 0.01, SE = 0.01, *P* = 0.917) had higher CMI:CRP ratio if they were born during the dry season compared to the wet (Fig. 4).

**Table 2.** Results from the multiple regression model of variation in CMI:CRP ratio in young adults from Cebu (*N* = 1248; *F*_12 1235 _= 7.95, Adj. *R*^2 ^= 0.063, *P* < 0001)

Variable	Estimate	SE	*P* value
Intercept	1.041	0.073	<0.0001
**Birth weight (kg)**	0.020	0.009	0.029
Weight gain, 0–2 years (kg)	0.005	0.004	0.198
Infectious morbidity, 0–2 years (no. of episodes)	0.001	0.002	0.779
**Likelihood of exposure to animal feces, 6–12 months (no. of intervals)**	0.007	0.003	0.023
**Maternal absence (0, 1)**	−0.027	0.013	0.048
Sibling death (0, 1)	0.005	0.012	0.672
Paternal instability (0, 1)	−0.011	0.014	0.432
Sex (female)	−0.060	0.009	-
Born in the dry season (0,1)	−0.008	0.013	-
**Interaction: born in the dry season (0, 1)** × **sex (female)**	0.065	0.019	0.001
Waist circumference, 2005 (cm)	−0.003	0.001	<0.0001
Total immune investment (CRP + CMI)	0.006	0.003	0.030

Weight gain during the first 2 years of life, the incidence of infectious morbidity, paternal instability, and sibling death were not significantly associated with adult CMI:CRP ratio (Table 2). Finally, individuals with greater waist circumference at the time of immune function assessment had a lower CMI:CRP ratio, while those with a greater overall immune score (CMI + CRP) had a higher CMI:CRP ratio (Table 2).

## Conclusions and Implications

In a sample of adults from Cebu, the Philippines, we showed that aspects of the prenatal and early post-natal environment were associated with variation in relative levels of investment in acquired versus innate immunity, as indicated by EBV antibody and CRP concentrations, respectively. Of the two variables measuring early nutritional condition (birthweight and weight gain during the first 2 years of life), only birthweight was significantly associated with adult immune phenotype. As predicted, individuals with higher birthweight had higher adult CMI:CRP ratio, suggesting greater investment toward acquired immune defenses. Of the three measures of early-life pathogen exposure (incidence of infectious morbidity, likelihood of exposure to animal feces, and being born in the dry season), two contributed to explaining the variance in adult CMI:CRP ratios, both in line with our predictions. First, individuals who experienced greater likelihood of exposure to animal feces in the first year of life showed higher CMI:CRP ratios. Second, among women but not among men, individuals born during the dry season (who spent a greater part of their first year during the wet season, a period of greater pathogen prevalence) exhibited higher CMI:CRP ratios. Finally, of the three measures of early-life psycho-social stress (maternal absence, paternal instability, and the death of a sibling), which can be seen as cues of extrinsic mortality, only one was significantly associated with adult immunophenotype. Individuals who experienced maternal absence in childhood showed lower adult CMI:CRP ratio, as predicted. In sum, these results provide partial support for the existence of trade-offs between the two arms of the immune system and indicate these trade-offs are affected by some early-life developmental factors, as predicted in our recent synthesis on this topic [31]. Although methodologically, the markers we employed to characterize the innate and acquired arms of the immune system (CRP and EBV antibody levels) have some important limitations as indicators of overall immunocompetence, our findings should be instructive for future attempts to examine how early life exposures may or may not affect adult immune function in humans.

The majority of ecoimmunology research on within-system trade-offs in immune development has thus far centered on relatively small-bodied and, by comparison to humans, shorter-lived species. Trade-offs between the arms of the immune system in such organisms are more likely to be pronounced, and indeed detected, as smaller animals do not have sufficient energy reserves to rely on as buffers/capital, in the way that larger-bodied animals do [62]. Nevertheless, by analyzing the factors affecting the ratio between our two measures of immune function, we showed that even relatively long-lived and large-bodied mammals such as humans are not exempt from such trade-offs. The prospective, longitudinal nature of our dataset allowed examining the effects of early environment on adult immune function on the extended time-frame of human maturation and development and thus highlights the benefits of adopting a long-term view when examining trade-offs within the immune system.

The importance of early-life pathogen exposure in potentially affecting levels of inflammation in young adulthood in this sample is already known: higher levels of pathogen exposure in infancy predicted reduced CRP in young Filipino adults [49], a finding that is consistent with a broader body of research linking more sanitary environments in infancy and early childhood with increased risk for immune-related diseases [63, 64]. Similarly, negative associations between birth weight and CRP have been documented in a range of populations, including in the present sample [49, 65, 66]. These associations have been interpreted primarily in mechanistic terms, with undernutrition acting as a constraint on immune development, and infectious exposures promoting the development of regulatory pathways that control inflammation. Recent studies have also documented positive associations between inflammation and maltreatment, harsh family environments, and socioeconomic adversity in childhood [67–72].

Existing data on inter-individual variation in measures of acquired immunity are also consistent with the logic of our framework [31]. Childhood adversity has been shown to predict elevated EBV antibodies in adulthood, indicative of reduced acquired immunity [73]. Undernutrition and lower levels of infectious exposure have also been associated with reductions in multiple aspects of acquired immunity [48,74–76]. The physiological and behavioral pathways linking early environments and adult human health are coming into focus [77], but an understanding of why these associations exist remains elusive. An evolutionary life history perspective points toward specific aspects of the developmental ecology as likely drivers of the immunophenotype, providing a framework for testing novel hypotheses regarding the causes and consequences of variation in multiple dimensions of human immunity.

Previous research in human ecological immunology has not explicitly tested the existence of trade-offs between the two branches of the immune system but some results are suggestive. Data from Cebu have shown the possible existence of such trade-offs between acquired and innate defenses: in a small subsample of subjects (*N* = 74), individuals that did not respond with antibodies to typhoid vaccination showed much higher CRP levels in adulthood [78]. Our current study, however, is the first to integrate measures of both arms of the immune system within a detailed developmental context across a much larger number of individuals (*N* = 1248). Our framework [31] thus extends previous findings by considering variation in one type of immune defense in the context of overall immune protection and suggesting that shifting priorities in resource allocation within the immune system might have adaptive significance. Incorporating a suite of predictors, relating to early life nutritional sufficiency, exposure to pathogens, and potential cues of mortality risk from the environment into a single analytical framework is a powerful approach to evaluate the relative contribution of each factor to the development of the immune system. Our results, indeed, suggest that both early ecological conditions (nutrition, pathogens) and factors that might affect the individual’s reproductive schedules (i.e. cues of extrinsic mortality) may have a long-term effect on the ratio of acquired to immune defenses.

The finding that season of birth affected the CMI:CRP ratios only in women could be either due to differences in exposure or to differences in sensitivity to exposure between the sexes. Although in some populations, male infants are raised under lower hygiene standards than female infants [79], whether this is also the case at Cebu is at present unclear. There are only marginal differences between male and female infants in their likelihood of exposure to animal feces (greater in boys: *P* = 0.06) and overall morbidity in the first 2 years of life (slightly greater in boys: *P* = 0.059, Table 1). Ongoing analyses suggest that boys may have significantly higher incidence of diarrhea in their first 2 years of life than girls (Kimberly McCabe, personal communication), but further immunological and ethnographic studies would be required to evaluate the possibility of gender-differentiated pathogen exposure.

Our final prediction that women would exhibit a relatively greater bias toward acquired defenses, given their longer life expectancy was not supported; in fact, females had significantly lower CMI:CRP ratios than men, suggesting a bias toward innate immune defenses. The finding that men also had greater levels of total immune investment (the sum of the standardized CRP and CMI values) than women is similarly unexpected as extensive existing data point toward women having a consistent immunological advantage over men [80–83]. Whether these patterns are due to the choice of markers, we used or whether they point to an overall greater investment toward acquired immune defense among men in this population is at present unclear. In particular, previous work has identified salient associations between inflammation (measured by CRP) and female reproductive function—with CRP differing by phase of the menstrual cycle, in relation to ovarian hormones, by whether the cycle was ovulatory or anovulatory, and in relation to women’s sexual activity [25, 84–90]. Although our examination of the effect of cycle phase (based on recall surveys) on women’s CRP value did not reveal significant effects within a subset of participants who exhibited regular recent cycling (unpublished data), female reproductive functioning may nevertheless explain the higher CRP levels among women, compared to men in our sample. Our reliance on CRP as the sole index of innate immunity may therefore be a methodological weakness that should be considered in the design of future studies.

Our analyses have focused on trade-offs between investments in innate and acquired immunity, but it is important to note that both systems are necessary for survival, and that in practice they work together to provide a relatively seamless network of defense. As such, one might expect small differences in relative levels of investment in relation to developmental environments. Indeed, the differences we find here are small and predictors in statistical models explain a tiny fraction of the variance CMI:CRP ratios (Table 2). This could partly be due to the fact that the most disadvantaged infants might have been lost from the sample due to early-life mortality and thus those who remained have only experienced relatively moderate adverse early exposures, thus reducing the explanatory power of predictors in the models. Previous research on this population, for example, has shown that infants with very low and high birthweight were more likely to die in the first week of life [91] so mortality-related attrition in this sample probably reduced the likelihood of finding significant relationships between early life conditions and adult immunophenotypes. The implications for morbidity, mortality, and other life history outcomes of the effects that we did identify in our analyses, however, are not clear. A large body of research suggests that increased inflammation contributes to cardiovascular disease, metabolic syndrome, dementia, and other diseases of aging [92–95], while deficiencies in acquired immunity are well-established determinants of infectious disease risk [96]. With subsequent follow up of the cohort, we will be able to evaluate the clinical significance of variable immunophenotypes.

It is also notable that when we examined the effects of early life exposures on total immune investment (CMI + CRP), none of the variables we considered had any explanatory power. This may be because early life exposures in this cohort were relatively benign and they did not reach a threshold beyond which they would have had an adverse effect on overall investment toward immune defenses. The weak trade-offs within the immune system we documented, however, suggest that some adjustment in allocation to resources within the immune system may occur even when overall ability to invest toward immune defenses is robust. This interpretation is consistent with the observed positive association between CMI:CRP ratio and total immune investment (CMI + CRP): individuals with greater overall investment in immune defenses prioritize acquired immunity over innate. Even small variation in resource availability may therefore be associated with shifts in the optimal trade-off level between acquired and innate defenses.

It is important to note that neither CRP nor EBV, measured on their own are sufficiently representative of innate and acquired immune function to provide us with definitive conclusions regarding trade-offs within the immune system. Both arms of the immune system comprised multiple pathways that interact in complex fashion. In addition to potentially being affected by female reproductive function, CRP levels may have also been related to subclinical inflammation processes that would not have been recognized from self-reported measures of morbidity. EBV, on the other hand, has been shown to be affected by current psycho-social stress [43], another factor we were not able to account for in our analysis. Our two markers are also limited in their longitudinal coverage, as they were both measured at a single time-point. However, our validation within a smaller subset of individuals from this sample (see Methods) showed that both measures used in this study exhibited some intra-individual stability over time and thus likely reflect some salient individual characteristics. Future work should consider a more comprehensive assessment of immunity, including functional measures of multiple dimensions of innate and acquired defenses evaluated under challenge or stimulation. In addition, our characterization of the nutritional, infectious, and psychosocial environment in infancy/early childhood is constrained by available variables. Despite these multiple sources of “noise” in the data that were available for this analysis, the prospective study design, with data collection beginning *in utero*, the wide range of available variables, and the large sample size, are significant strengths of this study. Our findings should nevertheless be regarded as preliminary, and generative of future tests and refinements of the proposed framework for within-immune-system trade-offs [31] based on research in diverse populations, across a range of ecological settings, and crucially, by using more sophisticated, multiple measures of immune function.
